# TiO2 nanoparticles induce cytotoxicity and genotoxicity in human alveolar cells

**DOI:** 10.1186/1755-8166-7-S1-P77

**Published:** 2014-01-21

**Authors:** Krupa Kansara, Pal Patel, Darshini Shah, NV Srikanth Vallabani, Ritesh K Shukla, Sanjay Singh, Ashutosh Kumar, Alok Dhawan

**Affiliations:** 1 Institute of Life Sciences, School of Science and Technology, Ahmedabad University, Ahmedabad –Gujarat, India

**Keywords:** Titania, Genotoxicity, Nanotoxicity

## Background

Engineered nanoparticles (ENPs) such as TiO_2_ are widely used in products such as cosmetics, clothing, food packaging, drug delivery systems, etc. due to their unique physicochemical properties. This has increased the liklihood of ENP exposure in humans. As the ENPs are having small size and high diffusion coefficient, they can migrate rapidly in the air. Therefore, inhalation is considered to be the primary route of exposure to such ENPs. Hence, in the present study an attempt was made to assess the potential toxicological effects of TiO_2_ NPs in human alveolar cell line (A549).

## Materials and methods

The average hydrodynamic size, size distribution, zeta potential and stability of TiO_2_ NPs in DMEM-F12 media were determined by dynamic light scattering (DLS). Internalisaiton of ENPs in cells was detected using flow cytometry. Cytotoxicity was assessed using the MTT and neutral red uptake (NRU) assays. The genotoxic potential of TiO_2_ NPs was assessed by cytokinesis block micronucleus (CBMN) assay and flow cytometry based assays.

## Results

The mean hydrodynamic diameter of TiO_2_ NPs in DMEM-F12 media, as measured by DLS was 23.27 ± 2.1nm and the zeta potential was -10.1 ± 1 mV. The particles were also found to be stable in the media for upto 72 hr. A significant (p<0.05) concentration dependent uptake of TiO_2_ NPs was obseverd as evident by an increase in the side scatter (SSC) intensity in flow cytomtery after 6 hr of exposure. A reduction in cell viability was observed as evident by the results of MTT and NRU both as a function of NP concentration as well as time of exposure. Moreover, significant (p<0.05) induction in the micronucleus formation was observed by conventional and flow cytometry based methods at non cytotoxic concentrations.

## Conclusion

Our data demonstrate that TiO2 ENPs are internalised in the human alveolar cells and induce cyto- and geno- toxicity. This warrant minimizing the unwanted exposure to the nanotechnology based products and suggests ensuring its safe use both by consumers and industry.

